# The Effects of Opioids on HIV Neuropathogenesis

**DOI:** 10.3389/fimmu.2019.02445

**Published:** 2019-10-18

**Authors:** Aniella Murphy, John Barbaro, Pablo Martínez-Aguado, Vanessa Chilunda, Matias Jaureguiberry-Bravo, Joan W. Berman

**Affiliations:** ^1^Laboratory of Dr. Joan W. Berman, Department of Pathology, Albert Einstein College of Medicine, Bronx, NY, United States; ^2^Laboratory of Dr. Joan W. Berman, Department of Microbiology and Immunology, Albert Einstein College of Medicine, Bronx, NY, United States

**Keywords:** HIV-associated neurocognitive disorders, substance abuse, buprenorphine, next generation sequencing, monocytes, macrophages, central nervous system

## Abstract

HIV associated neurocognitive disorders (HAND) are a group of neurological deficits that affect approximately half of people living with HIV (PLWH) despite effective antiretroviral therapy (ART). There are currently no reliable molecular biomarkers or treatments for HAND. Given the national opioid epidemic, as well as illegal and prescription use of opioid drugs among PLWH, it is critical to characterize the molecular interactions between HIV and opioids in cells of the CNS. It is also important to study the role of opioid substitution therapies in the context of HIV and CNS damage *in vitro* and *in vivo*. A major mechanism contributing to HIV neuropathogenesis is chronic, low-level inflammation in the CNS. HIV enters the brain within 4–8 days after peripheral infection and establishes CNS reservoirs, even in the context of ART, that are difficult to identify and eliminate. Infected cells, including monocytes, macrophages, and microglia, produce chemokines, cytokines, neurotoxic mediators, and viral proteins that contribute to chronic inflammation and ongoing neuronal damage. Opioids have been shown to impact these immune cells through a variety of molecular mechanisms, including opioid receptor binding and cross desensitization with chemokine receptors. The effects of opioid use on cognitive outcomes in individuals with HAND in clinical studies is variable, and thus multiple biological mechanisms are likely to contribute to the complex relationship between opioids and HIV in the CNS. In this review, we will examine what is known about both HIV and opioid mediated neuropathogenesis, and discuss key molecular processes that may be impacted by HIV and opioids in the context of neuroinflammation and CNS damage. We will also assess what is known about the effects of ART on these processes, and highlight areas of study that should be addressed in the context of ART.

## Introduction

People living with HIV (PLWH) now have much longer lifespans, and the incidence of AIDS related deaths has declined due to the advent of antiretroviral therapy (ART) and increased access to HIV care (1). Despite ART, HIV-1 (HIV) remains a major public health issue with 37 million people infected globally and over 38,000 new annual cases reported in the United States in 2017 (2). Currently, there is a major opioid abuse epidemic closely associated with HIV (3, 4). It is estimated that 2 million people in the United States have opioid use disorder (OUD), which is characterized by the abuse of prescription opioids and/or heroin that is commonly taken intravenously (4–6). Intravenous (IV) drug use is a major route for HIV infection. From the onset of the HIV epidemic, IV drug use accounted for approximately 36% of HIV cases in the United States (7). Thus, the risk of HIV infection and transmission is high among IV drug abusers. It has been shown that addiction to prescription opioids can lead to risky behaviors and injection drug use that facilitate HIV acquisition, indicating that the opioid epidemic may help to perpetuate the HIV epidemic (3, 8, 9). Not only does opioid abuse increase the risk for HIV infection, but also it is estimated that 20–50% of PLWH are prescribed opioids and are more likely to have OUD than their uninfected counterparts (10). Long term opioid use increases the risk of death in PLWH compared to such use in uninfected people (11). Thus, it is imperative to understand the unique relationships between opioids and HIV infection, and importantly, their roles in contributing to HIV-associated comorbidities.

One highly significant comorbidity is HIV associated neurocognitive disorders (HAND). It is estimated that 15–55% of HIV infected individuals will develop some form of HAND despite ART (12–15). HAND is a spectrum of neurocognitive deficits and impacts many cognitive domains, for which there is no treatment or prevention (15). It significantly impacts quality of life and other health outcomes in PLWH (15). HAND is diagnosed by a battery of neurocognitive testing. The results from this testing are classified into three categories, asymptomatic neurocognitive impairment (ANI), mild neurocognitive disorder (MND), and HIV associated dementia (16, 17). Pre-ART, HAD was a prevalent form of HAND. Due to increased access to ART and HIV care, HAD is seen infrequently in developed nations and most HAND diagnoses fall in the ANI and MND categories (14, 16). Yet, the prevalence of HAND has not changed significantly from the pre-ART era (15, 17). Although individuals with ANI do not manifest deficits in everyday cognitive function, evidence suggests that a diagnosis of ANI can transition to one of the more severe forms of HAND (14, 18, 19). It was shown that PLWH with ANI had an increased risk of cognitive decline compared to PLWH who were not cognitively impaired (18).

Currently, there are no therapies to eliminate HAND. Therefore, it is imperative to understand mechanisms that contribute to its development and severity. Evidence suggests that opioids impact cognitive function and outcomes in PLWH, indicating that they contribute to HAND pathogenesis (7, 20–25). PLWH who chronically use opioids have worse cognitive outcomes compared to PLWH who do not abuse these drugs (21). There is also an association between lifetime use of heroin with worse recall and working memory (21). These data highlight the consequences of opioid abuse in PLWH, and underscore that opioid abuse likely contributes to HAND.

Opioids may contribute to HAND by altering immune cell functions (26). The ability of opioids to affect the functions of cells critical to HIV neuropathogenesis, infection, and protection is being studied extensively. In this review, we will examine the mechanisms by which opioids impact HIV neuropathogenesis and the development of HAND.

## Effects of Opioids on Molecular Mechanisms That Mediate HIV Neuropathogenesis

### Mechanisms That Contribute to HIV Neuropathogenesis

HIV enters the CNS within 4–8 days after peripheral infection, establishing viral reservoirs in the brain most often before someone is aware of their HIV status (27, 28). These reservoirs are difficult to eliminate and persist despite effective ART, which is now prescribed at the time of detected seropositivity (27–29). The long-term consequences of taking ART daily on mechanisms that mediate HIV neuropathogenesis are largely unknown.

HIV enters the brain through the transmigration of a mature subset of monocytes that expresses CD14, the LPS co-receptor, and CD16, the FCγIII receptor, across the blood brain barrier (BBB) (30, 31). Monocyte transmigration is a multistep process in response to chemokines including CCL2 and CXCL12 (32–34). CCL2 and CXCL12 are potent monocyte chemoattractant proteins elevated in the brains of HIV infected individuals despite effective ART (33–35). They are produced in the CNS, translocated across the BBB, and presented on the surface of brain microvascular endothelial cells (BMVEC) (36, 37). The binding of CCL2 to CCR2, and CXCL12 to CXCR4 and/or CXCR7, facilitates the firm arrest of monocytes to the endothelium (38, 39). This is mediated, in part, by the binding of activated lymphocyte function-associated antigen 1 (LFA-1) and very late antigen 4 (VLA-4) to intercellular adhesion molecule 1 (ICAM-1) and vascular cell adhesion molecule 1 (VCAM-1), respectively (38–40). This process is followed by crawling and diapedesis of monocytes across the BBB into the CNS (41). Diapedesis occurs by homophilic interactions between junctional proteins of the Ig superfamily, including activated leukocyte cell adhesion molecule (ALCAM) and junctional adhesion molecule A (JAM-A), expressed on the surface of both monocytes and BMVEC (42–45). ALCAM and JAM-A are increased on the surface of HIV-infected mature monocytes, facilitating their transmigration across the BBB (46). The effects of ART and opioids on these steps have not been extensively examined.

In the brain, HIV-infected mature monocytes infect and activate other cell types such as macrophages, microglia, and astrocytes to a lesser extent, and can differentiate into long-lived perivascular macrophages (46–48). This differentiation replenishes HIV CNS reservoirs, contributing to the development of HAND. HIV-infected and activated cells also facilitate recruitment of additional monocytes into the CNS, establishing and propagating chronic neuroinflammation (34, 49–51). While neurons do not become infected with HIV, they are impacted by viral proteins, cytokines, and neurotoxins, all of which contribute to neuronal damage and loss and the development of neurocognitive impairment (48, 52–54). Table 1 summarizes the effects of opioids and ART on the various cell types that contribute to HIV neuropathogenesis.

**Table 1:** Effects of opioids, dopamine, antiretroviral therapy (ART), and buprenorphine on cellular functions that contribute to HIV neuropathogenesis.

	**Morphine**	**Dopamine**	**ART**	**Buprenorphine**
Monocytes	Increases adhesion to endothelium (55) Increases transmigration across the BBB (56–58)	Increases adhesion (59) Increases transmigration of uninfected cells across the BBB (60)	Not yet examined	Decreases adhesion to ICAM-1 on endothelial cells (61) Decreases chemotaxis to CCL2 (61, 62) Delays recycling of CCR2 to the cell surface (62) Decreases association of FROUNT with CCR2 (61)
Macrophages and Microglia	Increases HIV replication (63–69) May increase cytokine secretion (70–73) Increases ROS/RNS production (65, 73, 74)	Increases HIV replication (75, 76) Increases basal and LPS-mediated cytokine secretion in macrophages (77)	Increases ROS production in THP-1 derived macrophages (78)	Not yet examined
Astrocytes	Increases cytokine secretion (79, 80) Decreases glutamate uptake (79, 81) Increases glutamate release (82)	Outside the scope of this review	Increases IL-6 secretion (83) Decreases HIV-induced CCL2 and CCL5 secretion (84) Decreases glutamate uptake (85) Causes cellular senescence (83)	Not yet examined
T cells	May increase or decrease HIV replication (86–88) Decreases IL-2 secretion (89) Decreases T cell receptor signaling (89)	Outside the scope of this review	Not yet examined	Not yet examined

Another proposed mechanism that may contribute to HAND is immunosuppression due to HIV infection and chronic opioid usage. This can result in increased susceptibility to secondary infections and dissemination of bacteria into the CNS, increasing damage that causes neurocognitive dysfunction (26). These processes will not be discussed further as they are outside the scope of this review.

### HIV, Opioids, and Monocyte Transmigration Across the Blood-Brain-Barrier

Data both *in vitro* and in animal models suggest that opioids increase the transmigration of monocytes across the BBB in the context of HIV (55–58, 90, 91). Mice exposed intravenously to morphine and HIV Tat have increased numbers of inflammatory monocytes in the CNS compared to placebo mice and mice treated with Tat alone (58). Research in macaques demonstrated that exposure to morphine and infection with SIV increases the number of monocytes and macrophages in the brain, potentially contributing to neuropathogenesis (57). Findings from *in vitro* studies substantiated these results by demonstrating that morphine increases PBMC and monocyte adhesion to the endothelium that is further enhanced with exposure to gp120 (55, 91). Together, these data suggest that morphine exacerbates HIV neuropathogenesis by increasing monocyte entry into the CNS. Additional studies are needed to determine how morphine specifically increases uninfected and HIV-infected human monocyte transmigration, especially in the context of ART.

Opioids may increase monocyte transmigration by impairing integrity of the BBB (90–93). In human BMVEC, morphine and Tat decreased tight junction proteins, ZO-1, JAM-2, and occludin, leading to decreased transendothelial electric resistance and increased PBMC transmigration (90). Another group demonstrated that morphine treatment of BMVEC for up to 72 h increased ICAM-1 and VCAM-1, facilitating adhesion of PBMC to BMVEC monolayers (91). Although this group also found that morphine did not impair endothelial permeability to FITC-labeled dextrans, other evidence suggests that more chronic morphine exposure may increase BBB permeability (92, 94). Experiments using fluorescently labeled dextrans showed that transgenic mice expressing Tat have increased BBB permeability compared to control mice, and that morphine treatment for 5–7 days similarly increased permeability (94). Interestingly, this increased permeability did not correlate with increased penetration of antiretrovirals into the brain. Morphine treatment was shown to decrease antiretroviral concentrations in the brain by increasing P-glycoprotein (94). This finding is underscored by studies in rats demonstrating that morphine treatment for 5 days increases P-glycoprotein in the hippocampus and cerebral cortex (93). Thus, decreased penetration of ART into the CNS may also exacerbate HIV neuropathogenesis. The impact of opioids and ART together on BBB permeability, junctional protein expression, and adhesion of monocytes to the endothelium remain important to characterize in future studies.

Opioids increase dopamine concentrations in the CNS, contributing to their euphoric effects (95–97). Although dopamine does not cross the BBB to the periphery, the effects of increased extracellular dopamine on HIV neuropathogenesis are important to characterize because monocytes express surface receptors that bind dopamine as they cross the BBB (59, 60, 98). *In vitro* studies showed that dopamine and D1 receptor agonists increase monocyte adhesion, as well as transmigration of uninfected human CD14^+^CD16^+^ monocytes across a human BBB model (59, 60). These results suggest that monocyte influx is increased in regions of the brain that have increased dopamine concentrations (59, 60). This may be facilitated by increased active ADAM17, a metalloproteinase that cleaves the complex of Mac-1 on monocytes bound to endothelial ICAM-1 as they begin to diapedese (60). Primary human monocyte derived macrophages (MDM) also express dopamine receptors. Dopamine increases viral entry into these cells, resulting in increased viral replication (75, 76). In response to dopamine, human MDM also increase basal and LPS-mediated cytokine secretion (77). These data indicate that dopamine released in response to opioids or other drugs of abuse may increase monocyte migration into the CNS, and increase HIV infection and cytokine secretion by macrophages, contributing to HIV neuropathogenesis.

### HIV, Opioids, and the Roles of Microglia and Macrophages

Macrophages and microglia are phagocytic cells responsible for, among other functions, clearance of extracellular materials and production of cytokines and chemokines (99–102). In general, microglia are derived from the yolk sac and enter the CNS during embryonic development, while monocytes arise from hematopoietic progenitors, enter the brain throughout life, and can differentiate into long-lived macrophages (99, 100). There may be certain subsets of microglia that derive from the bone marrow as well (103, 104). Recent findings indicate that, within the CNS, macrophages and microglia have unique transcriptional profiles, suggesting that they may contribute to HIV neuropathogenesis by both similar and distinct mechanisms (105, 106). Some differentially expressed genes suggest that microglia modulate CNS neurotransmitter levels, while brain-resident macrophages promote toll-like receptor signaling and regulate growth factor concentrations and signaling (105).

Findings indicate that HIV reservoirs in microglia and macrophages still persist despite suppressive ART. Studies in animal models and PLWH taking ART have shown that HIV DNA and RNA colocalize with both macrophages and microglia in post-mortem brain tissue (107–109). Data from SIV-infected macaques on ART also demonstrated that brain macrophages in the CNS can still produce active virus, as shown using quantitative viral outgrowth assays (QVOA) (108). HIV-infected microglia and macrophages release neurotoxic viral and host factors, including HIV proteins, gp120, Tat, and Vpr, and reactive nitrogen/oxygen species (ROS/RNS), which damage neurons and activate other CNS cells, including astrocytes and endothelial cells (101, 110–113). Chronic disruption of CNS homeostasis leads to neurocognitive decline characteristic of HAND. Both macrophages and microglia are also activated by the microbial product, LPS, which is increased in the serum of PLWH even with ART (100, 110, 114). In response to LPS, microglia and macrophages increase production of TNFα, IL-1β, CCL2, and IL-8 that can damage neurons directly and increase transmigration of monocytes across the BBB (110, 112, 115). The mechanisms by which opioids contribute to these processes in microglia and macrophages in the context of ART are especially important to characterize such that they can be targeted therapeutically to reduce viral reservoirs and HAND.

Opioids may contribute to HIV-mediated CNS damage by regulating immune cell functions through opioid receptors. There are three classical opioid receptor subtypes, the μ (MOR), κ (KOR), and δ-opioid (DOR) receptors, all of which are expressed on the surfaces of microglia and macrophages (110, 116–118). There is a fourth, nociceptin receptor (NOP), that will not be discussed further in this review (119).

Results from many *in vitro* experiments demonstrated that morphine increases HIV replication in infected macrophages and microglia, although these results vary based on duration of opioid exposure (63–66). Increased viral replication may be due to a morphine mediated increase in galectin-1, as well as inhibition of anti-viral interferon signaling and microRNAs that target HIV genes for degradation (64–69, 110). Importantly, increased CCR5 expression in response to opioids also increases HIV replication in macrophages (63). This indicates that opioid mediated increased HIV replication is dependent on an HIV coreceptor that can be targeted therapeutically. Maraviroc is a known blocker of CCR5 that improves neurocognitive functioning in people with HAND (120, 121). It was also shown to decrease the number of CD16-expressing monocytes in PLWH, suggesting that maraviroc reduces the pool of infected monocytes in the periphery that can enter the CNS (121). Maraviroc may also reduce opioid mediated neuropathogenesis in PLWH by attenuating viral reservoirs in macrophages and microglia.

Although most studies found that morphine increases HIV replication, one study in MDM demonstrated that morphine may contribute to neuropathogenesis by increasing cytokines without affecting HIV replication (70). Other studies refuted this finding and showed no significant difference in cytokine production with opioids compared to HIV infection alone (71, 117). The mechanisms by which changes in viral replication may occur are important to characterize to reduce CNS viral load. Some studies suggest that this process is dependent on both MOR activation and on the subtype of MOR expressed by the cell (64, 110). While acute exposure to opioids has been shown to decrease intracellular cAMP through inhibitory G proteins, more chronic opioid exposure may increase cAMP (72, 102, 118). This increase leads to activation of proteins such as cAMP response element binding protein (CREB), which may bind to the 5′ LTR of the HIV genome to increase viral replication (66). Microglia and macrophages play critical roles as HIV harboring cells in the CNS. Thus, more studies are needed to address how opioid mediated changes in HIV replication occur in both cell types.

Chronic generation of reactive oxygen and nitrogen species has been implicated in the pathogenesis of HAND (110, 112). Productive HIV infection and/or exposure to HIV proteins, including Tat, increase ROS production in macrophages and microglia (69, 73, 110, 122). Additionally, multiple ART drugs, especially protease inhibitors (PI), including lopinavir and nelfinavir, and nucleoside reverse transcriptase inhibitors (NRTI), including zidovudine and stavudine, increase ROS in macrophages derived from THP-1 cells (78). The contribution of opioids to oxidative stress in the context of chronic HIV infection and/or exposure to neurotoxic HIV proteins, is complex (123). Studies in J774 murine macrophages showed that acute exposure to morphine decreases nitric oxide production, while morphine treatment of human MDM increases ROS through induction of certain miRNAs (65, 74). Findings in peritoneal macrophages demonstrated an increase in ROS when mice were given morphine twice daily for 5 days (117). Additional experiments showed that acute exposure to morphine and Tat synergistically increases ROS in murine microglia, an effect dependent on MOR (73). These results highlight that opioids may contribute to disease processes in the CNS by increasing oxidative stress in microglia and macrophages that harbor HIV or are exposed to HIV proteins. Given that PLWH and people taking pre-exposure prophylaxis (PrEP) are prescribed daily ART, it is important to examine how opioids affect oxidative stress in this context (124). This is especially relevant for macrophages, which differentiate from monocytes that have been exposed to higher levels of ART in the blood prior to transmigration into the CNS (100, 125).

Opioids may impact cytokine secretion in CNS macrophages and microglia, perpetuating neuroinflammation that contributes to neurocognitive decline. One study in murine microglia demonstrated that 24 h of morphine treatment decreases Tat mediated upregulation of IL-6, MCP-1, and TNF-α secretion, while another using primary human microglia showed that exposure of primary human microglia to morphine for 24 h increases IL-8 secretion but does not significantly change IL-6, MCP-1, or TNF-α secretion (69, 73). Few studies have addressed the effects of morphine on cytokine secretion in macrophages infected with HIV or exposed to HIV proteins (68, 70, 71). Future studies should determine the interactive effects of ART, opioids, and exposure to HIV on neuroinflammation propagated by macrophages and microglia. These may characterize molecular mechanisms by which microglia and macrophages contribute to neurodegeneration in both opioid abusers who are HIV-positive and those taking PrEP with the goal of developing therapeutics that reduce CNS inflammation and subsequent neuronal damage.

### HIV, Opioids, and Astrocytes

Astrocytes are the most abundant cells in the brain, and astrocyte homeostasis is essential for maintaining CNS function (126–129). Various studies demonstrated that astrocytes differ in morphology, suggesting varied functional capability (128, 130). Astrocytes regulate synaptic transmission through a well-characterized glutamate uptake mechanism, provide essential roles in synaptogenesis, regulate CNS ion homeostasis, produce ROS, and modulate CNS cells by responding to and producing chemokines and cytokines (126, 129, 131–133). Astrocytes also contact the endothelium to form part of the blood-brain-barrier (BBB), highlighting their importance in regulating monocyte entry into the CNS (134).

Astrocytes are less susceptible to productive HIV infection and may not effectively produce mature, infectious virions *in vivo* (107, 110). Early studies in post-mortem brain tissue demonstrated that HIV DNA and RNA colocalize with astrocytes, although more recent studies using tissue from PLWH who took ART did not find such colocalization (107, 135–137). Regardless of their infection status and ability to produce active virus, astrocytes produce and respond to neurotoxic viral proteins, gp120, Tat, and Nef (135, 137, 138). Exposure to these proteins leads to increased glutamate, nitric oxide, and cytokines that contribute directly to neuronal damage (79, 135, 137, 139). HIV infection of astrocytes increases Cx43, a protein that forms part of gap junctions and hemichannels, increasing delivery of neurotoxic factors and chemokines both directly to other astrocytes and to the extracellular space (140, 141). Upregulation of Cx43 colocalized with apoptosis of BBB cells in tissue from PLWH and SIV-infected macaques, which could contribute to a leaky BBB and enhance monocyte entry into the CNS (142).

Microarray and gene ontology studies showed that treatment of astrocytes with Nef induces expression of specific long non-coding RNAs (lncRNAs) that increase secretion of CXCL9, CXCL10, and CXCL11 (143). CXCL10 had already been shown to cause neuronal damage in response to Nef. These microarray and gene ontology data suggest that CXCL9 and CXCL11 secreted by astrocytes may also be important in HIV neuropathogenesis (143). Astrocytes treated with Tat and co-cultured with microglia increased release of intact exosomes containing miR-9, a miRNA that was taken up by microglia and stimulated their migration *in vitro* (144). Recent findings also demonstrated that astrocytes exposed to HIV, but not productively infected, have increased mitochondrial damage and ROS, activating NLRP3 inflammasomes and increasing IL-1β secretion (145). Conversely, astrocytes harboring HIV cleared damaged mitochondria, leading to decreased ROS and IL-1β. These data emphasize that even uninfected astrocytes can contribute to HIV neuropathogenesis through increased ROS and cytokine expression.

Astrocytes may play a significant role in opioid mediated neuropathogenesis in the context of HIV given their abundance and expression all three opioid receptor subtypes (MOR, KOR, DOR) (146–148). Opioids can exacerbate CNS damage through disruption of normal astrocyte functions (80, 81, 147, 148). Astrocytes are critical to regulating local synaptic glutamate concentrations, and increased synaptic glutamate leads to neuronal excitotoxicity (126, 135, 137). Opioids decrease astrocytic expression of glutamate transporters, GLT-1 and GLAST, which are essential for glutamate uptake (81). Recent studies demonstrated that opioids also increase glutamate release from astrocytes through specialized potassium channels, a process dependent on Gαi-coupled GPCR activity (80). Morphine has also been shown to decrease glutamate uptake in astrocytes infected with HIV or exposed to HIV proteins (79). These findings suggest that opioids may increase CNS damage by furthering disruption of astrocytic functions already impaired in the context of HIV (148).

The interactive effects between HIV and opioids on cytokine production in astrocytes are complex (79, 80, 84). While morphine by itself has minimal effects on cytokine secretion, astrocytes treated with morphine and Tat secreted more IL-6, CCL2, and TNF-α compared to cells treated with Tat alone. HIV-infected astrocytes treated with morphine produced more CCL2, IL-8, and TNF-α compared to astrocytes infected with HIV but not treated with morphine (79, 80). This increase in cytokines may enhance local inflammation that damages neurons, recruits additional infected cells from other regions of the CNS, and promotes further monocyte transmigration across the BBB. Transcription of these cytokines is regulated by NFκB, which is activated downstream of MOR (139, 149, 150). Productively infected primary human astrocytes also express TLR4 and produce cytokines in response to pathogen associated molecular patterns (PAMPs) like LPS (138, 139, 151). Given that morphine itself may activate TLR4, agonism at this receptor could act synergistically with MOR to increase NFκB mediated inflammatory cytokines (152).

Crosstalk between chemokine and opioid receptors in astrocytes may also contribute to opioid mediated HIV neuropathogenesis (132, 133). In neuron-astrocyte co-cultures, expression of CCR5 by astrocytes was shown to mediate opioid driven exacerbation of Tat induced neuronal damage (153). When CCR5 was deleted from glial cells or blocked pharmacologically with maraviroc, morphine treatment resulted in a surprising neuroprotective effect in response to Tat (153). These results emphasize that the impact of opioids on neuronal damage during HIV infection may change based upon chemokine receptor signaling. These data also suggest that maraviroc may improve neurocognitive outcomes in PLWH with OUD through its effects on astrocytes in addition to monocytes and macrophages (120, 121).

Potential mechanisms that mediate astrocyte dysfunction in the context of ART remain important to characterize. Some studies examined the effects of ART on astrocyte function, although few have addressed effects on HIV neuropathogenesis during exposure to opioids and ART together (83–85). One study using a combination of ritonavir, abacavir, and lamivudine demonstrated that treatment of astrocytes for 7 days leads to a cellular senescent phenotype characterized by increased cell cycle inhibitor, p21, increased oxidative stress, and increased IL-6 (83). Another study demonstrated that the PIs, amprenavir and lopinavir, decrease glutamate transporter, EAAT2, exacerbating defects in glutamate uptake (85). Others examined the effects of opioids and ART together on viral replication and cytokine expression in astrocytes infected *in vitro* (84). While a combination of the NRTI, emtricitabine, and two PIs, ritonavir and atazanavir, effectively reduced p24 production over 7 days, this effect was attenuated when astrocytes were co-incubated with morphine (84). This ART combination also inhibited CCL5 and CCL2 production in HIV-infected cells. However, when HIV-infected cells were treated with ART and morphine, they produced more CCL5 and CCL2 than infected cells that did not receive ART or morphine (84). These findings indicate that ART drugs may disrupt astrocyte homeostasis, underscoring the need to study the effects of ART in the presence and absence of opioids on the contribution of astrocytes to neuropathogenesis in PLWH.

### Opioids, HIV, and the Potential Role of T Cells

T cells play a significant role in many inflammatory processes in the CNS (48, 154). While *in vitro* evidence for a role of T cells in HIV neuropathogenesis is limited, some clinical studies suggest that T cells contribute to HIV neuropathology (111, 155–157). Two studies of brains from HIV-positive individuals demonstrated the presence of CD8+ T cells (155, 157). However, these studies were done in the context of HIV-encephalitis (HIVE) and HIV-associated dementia (HAD), which are not as prevalent as milder forms of HAND in the ART era (155, 157). There is also a significant correlation between nadir CD4+ T cell level and neurocognitive impairment in PLWH, but this association could indicate more generally severe systemic disease, and does not substantiate direct T cell contribution to neuropathogenesis (156). A population of CD8+ T cells is increased in the CSF of PLWH with cognitive impairment (158, 159). One study demonstrated that CD8+ T cells present in the CSF of PLWH have increased VLA-4 and CXCR3, while another found that levels of IFN-γ in these CSF CD8+ T cells correlated with severity of neurocognitive impairment (158, 159). These results suggest that the inflammatory signature of T cells in the context of HIV neuropathogenesis may be distinct and could perhaps be targeted therapeutically (158). Release of factors such as IFN-γ from T cells may be indicative of a compensatory anti-viral response that inadvertently promotes activation and polarization of M1 macrophages to further neuroinflammation. However, the contribution of T cell transmigration across the BBB to development of HAND is less clear (110).

In most cases, opioids have immunosuppressive effects in T cells (89, 160). In fact, heroin-addicted individuals have increased regulatory T cells (Tregs) in peripheral blood compared to healthy controls or to opioid abusers currently undergoing opioid substitution therapy (161). While little is known about the contribution of opioids to the potential roles of T cells in HIV neuropathogenesis, some studies have examined the effects of opioids on T cell functions (89, 160–162). Opioids can impact T cell functions by binding surface MOR, KOR, and DOR (86, 87, 160). Opioid receptor activity has been shown to inhibit T cell proliferation and activation through suppression of IL-2 secretion and T cell receptor signaling, and exacerbate cell death induced by gp120 (89, 163). All of these may contribute to systemic immunosuppression characteristic of chronic opioid abuse and increase susceptibility to infection (72). Both KOR and DOR activity have been shown to inhibit production of HIV in CD4+ T cells, and opioid receptor activity does not seem to play a role in reactivation of HIV in latently infected cells (86, 87, 163). However, naltrexone treatment of CD4+ T cells, which blocks opioid receptors, may potentiate the antiviral effects of ART drugs, such as AZT and indinavir. Thus, it is necessary to study the effects of opioids on T cell functions in the context of ART (88). For CD8+ T cells, which may be increased in the CNS, morphine has been shown to inhibit IFN-γ dependent anti-HIV activity (164). These results indicate that T cells in the HIV-infected CNS exposed to opioids may be limited in their capacity to control CNS viral replication, exacerbating CNS damage (88, 161, 162, 164, 165). Further studies are needed to determine how opioids, opioid substitution therapy, and ART regulate T cell activation and suppression to impact CNS damage in PLWH with OUD.

## Opioid Substitution Therapy

One way to treat opioid addiction is through opioid substitution therapy (OST), including methadone and buprenorphine (166). Currently, little is known about the molecular impacts of these therapies and specifically their contribution to HIV neuropathogenesis in the ART era.

Methadone and buprenorphine are two of the most commonly used OSTs (167). Methadone is a synthetic opioid and full MOR agonist with long lasting effects (168). Its pharmacological actions are similar to morphine (168). Buprenorphine is a semi-synthetic opioid, long lasting partial agonist of MOR and a full antagonist of KOR (169). While both methadone and buprenorphine are effective at treating opioid dependence, some studies suggest these treatments may differentially impact cognitive outcomes in people with OUD. Buprenorphine treated drug abusers had improved cognitive outcomes compared to baseline or those treated with methadone (170–172). However, other studies found no difference in cognitive performance between methadone and buprenorphine treated opioid users (173). Overall, these studies were done with small sample sizes. Studies with larger numbers of subjects are needed to address definitively whether one therapy has improved outcomes compared to the other. A few studies also compared cognitive outcomes of drug users taking buprenorphine or methadone to healthy controls (171, 174, 175). In these studies, both buprenorphine treated and methadone treated individuals performed worse in multiple cognitive domains than their healthy counterparts (171, 174, 175). The healthy controls were individuals who did not have OUD or a history of chronic drug use. It is important note that individuals who are taking methadone or buprenorphine for OUD may already have cognitive deficits due to chronic drug use (12, 21, 25). A healthy individual who does not chronically abuse drugs may not manifest the same deficits as an individual who does. While comparing cognitive outcomes among these groups will address their effects on normal cognition, it may mask positive effects of buprenorphine and methadone on cognitive outcomes in people with OUD.

Some evidence suggests that PLWH are at higher risk for OUD than their uninfected counterparts (10). Thus, it is important to understand the effects of OST in the context of HIV neuropathogenesis and ART. Few studies have directly addressed the impact of methadone or buprenorphine on HIV-mediated CNS damage. Both methadone and buprenorphine reduce illicit opioid use and risky behaviors associated with HIV infection (176). In some cases, individuals taking methadone or buprenorphine were more likely to initiate or remain on ART and have improved CD4+ T cell counts (20, 22, 176). This demonstrates that OST can successfully treat opioid dependence and improves both the health and quality of life of PLWH who have OUD (22, 171, 177–181). However, in one study, HIV infected men taking methadone performed worse on cognitive tests than those who were not taking methadone (182). In contrast, some evidence suggests that PLWH taking buprenorphine have improved quality of life and neuropsychological functioning (12, 179, 183). Thus, buprenorphine appears to be a promising therapy in the context of HIV mediated CNS damage. More studies are needed to evaluate the effects of both methadone and buprenorphine on cognitive outcomes in PLWH, especially in the context of the current guidelines to initiate ART at the time of diagnosis.

**Figure 1:**
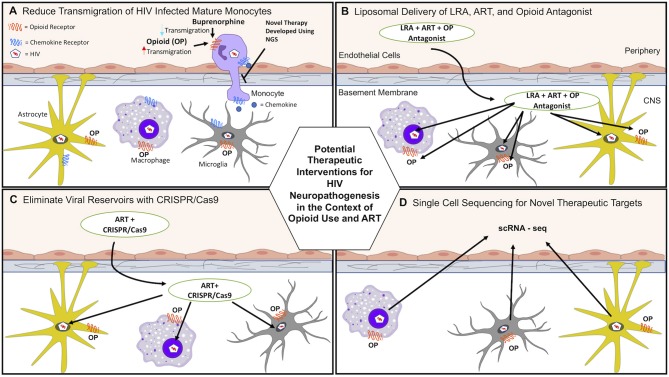
Potential therapeutic interventions for HAND in the context of opioid use and antiretroviral therapy (ART). **(A)** HIV induced neuroinflammation and viral seeding can be reduced by decreasing monocyte transmigration into the brain that is increased with opioid abuse. Buprenorphine and/or novel therapies developed using next generation sequencing (NGS) may reduce transmigration of HIV-infected and uninfected monocytes. **(B)** Current ART penetration into the brain does not eradicate viral reservoirs. Latency reactivating agents (LRA), ART, and opioid antagonists can be transported across the BBB and target infected cells in the CNS using liposomal nanoparticle delivery systems. **(C)** ART and CRISPR/Cas9 can be transported across the BBB to help eradicate HIV reservoirs using liposomal nanoparticle delivery systems. **(D)** scRNA-seq can be used to identify novel molecular mechanisms by which opioids and HIV infection increase CNS damage. These mechanisms can then be targeted to reduce HAND.

Data from our laboratory propose that buprenorphine may decrease HIV neuropathogenesis through its effects on monocytes, and underscore the potential therapeutic effects of buprenorphine as a treatment for HAND (61, 62). Buprenorphine decreased important steps in the transmigration of mature monocytes across the BBB, including CCL2-mediated adhesion to ICAM-1 and chemotaxis (Table 1) (61, 62). These results indicate that buprenorphine may reduce HIV neuropathogenesis and cognitive impairment by decreasing monocyte entry into the brain (Figure 1A, Table 1).

The mechanisms by which buprenorphine mediates these effects are unclear. One reason may be due to its unique pharmacological properties (169). Buprenorphine is a partial MOR agonist with high affinity for the receptor but low intrinsic activity (166). This is distinct from other opioids such as morphine, codeine, fentanyl, and methadone that are full MOR agonists (169, 184). Full agonists and partial agonists may have different binding properties that facilitate unique signaling responses. This process is called biased agonism, and occurs when different ligands bind the same receptor but activate different intracellular signaling pathways.

Both opioid receptors and chemokine receptors, including CCR2 and HIV co-receptors, belong to a family of G-protein coupled receptors (GPCRs) that signal through the inhibitory Gα protein, Gαi. GPCRs are downregulated by desensitization, one form of which is heterologous desensitization (185, 186). During heterologous desensitization, activation of one GPCR inactivates signaling from a different GPCR (185, 187, 188). This may be one mechanism by which buprenorphine impacts monocyte function. Buprenorphine may inhibit downstream inflammatory signaling of chemokine receptors like CCR2 through its actions at MOR and KOR (62). We showed that buprenorphine decreases the association of FROUNT with CCR2 (62). This association mediates CCR2 signaling and enhances CCR2 induced monocyte transmigration (189). Thus, buprenorphine may decrease monocyte transmigration into the brain by limiting the interaction of FROUNT with CCR2.

Buprenorphine may also regulate GPCR signaling through heterodimers consisting of one opioid receptor subunit and one chemokine receptor subunit, resulting in desensitization upon simultaneous binding of opioids and chemokines to their receptors (5). It has been shown that DOR can heterodimerize with CXCR4 (190). Concomitant treatment with buprenorphine and a chemokine may result in heterodimers of opioid and chemokine receptors, thus affecting the signaling of each receptor subunit (191, 192). This may also affect the transmigration of mature monocytes across the BBB to contribute to HAND. More studies are needed to characterize the effects of buprenorphine on transmigration of monocytes and other immune cells *in vitro* and *in vivo*, as well as to address the impacts of methadone and buprenorphine on cognitive functions in PLWH, especially in the context of OUD and ART.

## New Technologies and Potential Therapies

New developments in molecular, genetic, computational, and next generation sequencing (NGS) techniques provide exceptional opportunities to characterize mechanisms by which opioids and HIV infection in the ART era impact neuroinflammation and neuronal damage, and may ultimately lead to development of therapies. Figure 1 illustrates novel approaches for identifying mechanisms as well as potential therapeutic interventions to reduce the burden of HIV neuropathogenesis in the context of OUD. Table 2 summarizes new technologies and therapeutic strategies to target HIV mediated CNS damage in the context of opioid use.

**Table 2:** Novel approaches for targeting processes that contribute to HIV neuropathogenesis in the context of opioid use.

**Approach**	**Cell type or mouse model**	**References**	**Current and/or potential application(s) to opioid and HIV mediated neuropathogenesis**
X-ray crystallography	Human delta-opioid receptor (δ-OR)	(193)	Design new ligands with fewer opioid induced side effects
Computational structure-guided molecular docking approaches	Newly synthesized opioid receptor ligands	(194, 195)	Design and synthesize novel opioid receptor ligands to reduce HIV mediated neuropathogenesis in the context of opioid abuse
SELEX, CLIP	Protein-RNA complexes	(196–199)	Develop chemical inhibitors of HIV proteins
Optogenetics	Chimeric photosensitive MOR-like receptors	(200)	Activate μ-opioid receptors in specific CNS cells to study their effects on behavior and neurocognition
CRISPR/Cas9	Microglial cell line	(201)	Evaluate therapies for HIV-mediated neuroinflammation and neurodegeneration during opioid use
Mouse models	JR-CSF/hCycT1 hCD4/R5/cT1 Humanized myeloid only mice BLT mouse	(202–205)	Characterize *in vivo* the impact of opioids on HIV mediated neuroinflammation in the context of ART, and determine the efficacy of potential treatments
Genetically modified viruses	EcoHIV	(16, 206)	Examine the effects of opioids and HIV infection on cognitive deficits, including in genetically modified animals
Nanotechnology(+CRISPR/Cas9)	PBMC BBB Model Microglia	(207, 208)	Target both latent HIV and the effects of opioid abuse by combining drugs in one nanoformulation
Bivalent Ligands	Astrocytes	(209)	Inhibit viral entry by targeting opioid and chemokine receptor heterodimers
RNA-seq	PBMC	(210)	Identify mechanisms by which PBMC are impacted by HIV infection and opioid use
Single cell RNA-seq	Oligodendrocytes Myeloid cells Single brain nucleus	(211–213)	Characterize the effects of HIV and opioids on different cell types, and determine the impacts of opioids and HIV on gene expression in the CNS using frozen tissue

### Novel Technologies to Characterize Opioid Receptor Function

Structural and functional characterization of opioid receptors is especially important for understanding signal transduction after ligand binding. Advances in high resolution x-ray crystallographic techniques have led to crystal structures for all four known opioid receptors, including structures of receptors with associated peptides (193). These advancements are pivotal to develop new opioid receptor ligands with increased therapeutic actions and fewer side effects (Table 2). For example, structure-guided molecular docking approaches enable screening of millions of candidate compounds by computational studies, leading to the synthesis and optimization of selected molecules with novel biological effects on opioid receptors (194, 195). These studies could lead to development of compounds that reduce neuropathogenesis caused by morphine induced cytokine secretion, increased viral replication, and other important functions of microglia, macrophages, and astrocytes.

Multiple isoforms of opioid receptor subtypes can be generated by alternative splicing, and HIV infection has been suggested to play a role in promoting formation of these isoforms. One study found that an increase in the MOR-1K isoform correlates with severity of neurocognitive impairment in PLWH (214). The mechanisms by which HIV mediates these alterations in splicing remain uncharacterized, but modifications in several specific RNA-binding proteins may be involved (215). Selected evolution of ligands through exponential enrichment (SELEX) and cross-linking and immunoprecipitation (CLIP) have been used to characterize the role of these proteins (196, 197). Understanding these mechanisms led to screening of chemical inhibitors developed to inhibit key HIV regulatory proteins (198, 199) (Table 2). Therefore, a more extensive characterization of how HIV can impact the pharmacology of individual opioid isoforms is essential to understand how opioids contribute to HAND in the context of ART.

Another technology is optogenetics. Optogenetics is an experimental approach that modulates neural activity by activating and inhibiting light-sensitive proteins (216). Advances in optogenetics led to generation of chimeric photosensitive MOR-like receptors. This enables researchers to study the spatiotemporal control of opioid receptor signaling *in vitro* and *in vivo*. It was shown that *in vitro* photostimulation of cultured murine dorsal root ganglia neurons resulted in similar signaling and receptor internalization to that of the unmodified μ-opioid receptor. Using this approach *in vivo*, this group also found that MOR-like receptor signaling can be modified with photostimulation, resulting in behavioral responses similar to those observed when stimulating the unmodified receptor (200). This technology could be used in HIV-infected animal models to activate MOR in specific CNS cells types and study effects on behavior and cognitive status (Table 2).

### Genome Editing Technologies

Novel genome editing technologies have led to new models to study opioid mediated HIV neuropathogenesis. One study used the CRISPR/Cas9 system to integrate a modified HIV-provirus expressing a nanoluciferase reporter into the genome of a microglial cell line. This enables rapid assaying of HIV proviral activity induced by pharmacological manipulation, and would be useful to evaluate therapies for HIV-associated neuroinflammation and neurodegeneration in the context of opioid use (Table 2) (201). Mouse models have also been developed to study HIV neuropathogenesis. The JR-CSF/hCycT1 is a transgenic mouse that produces HIV, leading to plasma viremia. This model is particularly useful to study the pathogenesis of HIV-mediated neuroinflammation because these mice express a full-length HIV provirus virus that replicates in CD4^+^ T cells and monocytes (202). Another transgenic mouse model, the hCD4/R5/cT1, has been modified to enable acute *in vivo* HIV infection after intravenous, intravaginal, and intrasplenic inoculation, resulting in local infection and plasma viremia (203). Thus, these models provide opportunities to study the impact of opioids on HIV replication and inflammation in the context of ART *in vivo*, and to determine the efficacy of potential therapies. Additional new approaches include genetically modified viruses, such as EcoHIV, which is used to infect mice as a model for studying chronic HIV CNS disease (16, 206). This model has a low level of infection and develops neurocognitive impairment. It can also be used to study the effects of opioids and HIV infection on cognitive deficits in genetically manipulated mice (206). Other model murine systems include humanized myeloid-only and BLT mice (204, 205) (Table 2).

### Novel Therapeutic Approaches

Current ART does not eradicate HIV CNS reservoirs or improve the complications of HIV infection in the context of opioid abuse. One strategy involves using encapsulated liposomal magnetic nanoparticles containing a combination of ART drugs to prevent HIV replication, latency reactivating agents to activate CNS viral reservoirs, and drug abuse antagonists to ameliorate associated side effects. These novel nanoparticles have several advantages. Different drugs can be combined in a single nanoformulation to target simultaneously latent HIV and opioid induced CNS damage (Table 2). Additionally, they can be targeted to a specific tissue by non-invasive magnetic forces applied at adjustable intensities. They can also cross the BBB, avoiding a major restriction of the transport of many ART drugs (Figure 1B) (207).

Although nanotechnology is a promising approach to treat opioid associated neuropathogenesis in PLWH, there are few *in vivo* studies showing site specific targeting for antiretrovirals and drug abuse antagonists. Thus, BBB transmigration efficiency, appropriate concentrations of drug to be added, and magnetic treatment parameters have to be optimized for human application.

Another nanotechnology-based approach includes gene editing tools to eradicate HIV brain reservoirs in drug abusers. CRISPR/Cas9 technology is being used to eliminate latent HIV from the genome by specifically targeting the enhancer of the HIV LTR promoter (217). However, this technique still requires an effective method for delivery across the BBB. To overcome this obstacle, it has been combined with magnetic nanodelivery systems, showing a significant HIV-LTR reduction, indicating a potential therapy to eliminate latent HIV infection in the brains of opioid abusers (Figure 1C) (208).

Structural and functional characterization of MOR-CCR5 heterodimers may help to study synergistic effects of HIV infection and opioid abuse in the CNS, as discussed earlier in this review. One study used novel synthesized bivalent ligands, combining a MOR antagonist and a CCR5 antagonist into one molecule, to target these heterodimers in HIV-infected astrocytes, inhibiting HIV entry two-fold compared to a mixture of individual MOR and CCR5 antagonists (Table 2) (209).

### Next Generation Sequencing Technologies

RNA sequencing (RNA-seq) studies have been used to analyze transcriptional profiles of different brain regions in the context of long-term opioid abuse leading to neurodegeneration. Chronic oxycodone self-administration in mice was shown to increase expression of genes related to inflammation and/or immune function, including CCR5, in the dorsal and ventral striatum (218). Increased CCR5 expression facilitates migration of cells to sites of inflammation in response to chemokines, increasing their susceptibility to infection (7, 63, 64, 219, 220). RNA-seq studies have also been performed with cells from human cohorts. One study in PBMC from long-term heroin users showed upregulation of genes related to neurological and psychiatric disorders, including spinocerebellar ataxia type 2 and posttraumatic stress disorder (210) (Table 2). These cells can enter the CNS, underscoring the importance of using new tools to understand mechanisms by which they contribute to neuropathogenesis related to opioid abuse.

The brain is a heterogeneous organ, and therefore RNA-seq analysis may not fully identify and assign unique gene expression profiles to specific cells in each brain region. To overcome this limitation, single cell transcriptome analytic approaches can be used. Single cell RNA sequencing (scRNA-seq) is a powerful tool that can be used to address many questions relevant to HIV infection and OUD (Figure 1D). It is of particular interest to determine how different cell types in brain regions are affected by chronic opioid exposure. A recent study demonstrated that acute morphine treatment of mice results in unique transcriptional responses by oligodendrocytes, suggesting a link between opioid abuse and myelin dysregulation (211).

ScRNA-seq analysis can characterize how HIV impacts individual CNS cell types by modifying their gene expression. One study performed this analysis on cells obtained from CSF and blood from adults with and without HIV infection, and identified a rare subset of myeloid cells present only in CSF (Table 2). The gene expression profile of these cells showed significant similarities to the profiles of neurodegenerative disease–associated microglia (212). scRNA-seq can also be used to identify the molecular characteristics of CNS and peripheral blood cell types that harbor HIV during opioid abuse. Our group found that CD14^+^CD16^+^ monocytes that specifically harbor HIV preferentially transmigrate across an *in vitro* human BBB model in response to chemokines compared to uninfected monocytes exposed but not harboring the virus (46, 221). Thus, the identification of gene expression profiles of individual HIV-infected cells may provide new strategies to eliminate HIV in the CNS of opioid abusers. However, although scRNA-seq is widely used to study individual cell transcriptomes, isolation of single cells from complex tissues such as the brain is difficult. Newly improved single cell sequencing techniques, such as single-nucleus RNA sequencing, enables analysis of gene expression within a single brain nucleus, reducing cell biases caused during cell isolation and facilitating the use of frozen samples (213).

Next generation sequencing technology, and especially single cell genomics, will be important tools for the molecular characterization of opioid abuse in the context of HIV infection and ART. Studies using these technologies may lead to identification of novel therapeutic targets and diagnostic biomarkers, enabling early intervention to improve neurocognitive outcomes in PLWH with OUD.

## Conclusion

HIV remains a significant worldwide health issue as does the relationship between HIV infection and the opioid epidemic. HAND persists despite successful ART, for which there are currently no treatments. Ultimately, the combined effects of opioid use and HIV infection have negative consequences on neurocognitive outcomes. Thus, it is important to understand the mechanisms by which opioids contribute to HIV mediated CNS damage, especially in the context of suppressive ART. Opioids may contribute to HIV neuropathogenesis through regulation of immune cell functions. The specific mechanisms by which opioids and HIV infection interact remain unclear. Buprenorphine appears to have beneficial effects on cognitive outcomes in PLWH. More studies are needed to examine how opioids and OST contribute to or limit HIV neuropathogenesis. Now that PLWH are prescribed ART at the time of diagnosis. Current and new technologies will characterize how opioids impact HIV infection of the brain in the ART era, and will direct the development of novel therapies for HAND in PLWH with OUD.

## Author Contributions

AM, JB, PM-A, VC, and MJ-B contributed to discussions about manuscript content, writing, and editing of the manuscript. JWB contributed to discussions of the manuscript and its editing.

### Conflict of Interest

The authors declare that the research was conducted in the absence of any commercial or financial relationships that could be construed as a potential conflict of interest.

## Abbreviations

HAND: HIV associated neurocognitive disorder
PLWH: People living with HIV
OUD: Opioid use disorder
OST: Opioid substitution therapy
ICAM-1: Intracellular Adhesion Molecule 1
VCAM-1: Vascular Adhesion Molecule 1
LFA-1: Lymphocyte lymphocyte function-associated antigen 1 (LFA-1)
VLA-4: Very late antigen 4 (VLA-4)
JAM-A: Junctional adhesion molecule A
SIV: Simian immunodeficiency virus
CNS: Central nervous system
BMVEC: Brain microvascular endothelial cells
JAM-2: Junctional adhesion molecule 2
ALCAM: Activated Leukocyte Cell Adhesion molecule
ANI: Asymptomatic neurocognitive impairment
MND: Mild neurocognitive disorder
HAD: HIV associated dementia
ART: Antiretroviral Therapy
BBB: Blood brain barrier
ADAM17: A disintegrin and metalloproteinase 17
MDM: Monocyte derived macrophages
MOR: μ-opioid receptor
KOR: κ-opioid receptor
DOR: δ-opioid receptor
NOP: Nociceptin opioid receptor
ROS: Reactive oxygen species
RNS: Reactive nitrogen species
PI: Protease inhibitor
NRTI: Nucleoside reverse transcriptase inhibitor
lncRNAs: long non-coding RNA
GPCR: G protein coupled receptors
HIVE: HIV encephalitis
CSF: Cerebrospinal fluid
NGS: Next generation sequencing
SELEX: selected evolution of ligands through exponential enrichment
CLIP: cross-linking and immunoprecipitation
RNA-seq: RNA sequencing
PBMC: Peripheral blood mononuclear cells
scRNA-seq: single cell RNA sequencing
ZO-1: Zonula occludens 1
QVOA: Quantitative viral outgrowth assay
LPS: Lipopolysaccharide
CREB: Cyclic AMP response element binding protein
LTR: Long terminal chain repeat
NLRP3: NLR family pyrin domain containing 3
GLT-1: Glutamate transporter 1
GLAST: Glutamate aspartate transporter 1
TLR: Toll-like receptor
PAMP: Pathogen associated molecular patterns
AZT: zidovudine
LRA: Latency Reactivating Agents
OP: Opioid
PrEP: Pre-exposure prophylaxis.
